# Metagenomic Insights into Microbial Community Structure, Function, and Salt Adaptation in Saline Soils of Arid Land, China

**DOI:** 10.3390/microorganisms10112183

**Published:** 2022-11-03

**Authors:** Jianjun Yang, Wenjing Li, Dexiong Teng, Xiaodong Yang, Yijun Zhang, Yan Li

**Affiliations:** 1 College of Ecology and Environment, Xinjiang University, Urumqi 830046, China; 2 Key Laboratory of Oasis Ecology, Ministry of Education, Urumqi 830046, China; 3 Xinjiang Jinghe Observation and Research Station of Temperate Desert Ecosystem, Ministry of Education, Urumqi 830046, China; 4 Institute of Applied Ecology, Chinese Academy of Sciences, Shenyang 110016, China; 5 Department of Geography & Spatial Information Technology, Ningbo University, Ningbo 315211, China

**Keywords:** metagenomics technology, soil salinization, hypersaline, KEGG annotation, adaptation strategy, osmolyte regulation, ion transport

## Abstract

Soil salinization is spread in the dryland of NW China due to the dry or extreme dry climate. Increased salinization damages the health and function of soil systems and influences the microbial community structure and function. Some studies have been conducted to reveal the microbial community structure and isolate the microorganisms of saline soil or salt-lake sediments in this region. However, the functions of microorganisms and their response to salinization, i.e., their adaptation strategy to a wide salinization range in arid environments, are less understood. Here, we applied metagenomics technology to investigate the microbial community structure, function, and their relationship with salinization, and discussed the adaptative strategy of microorganisms to different saline environments. A total of 42 samples were sequenced on the Illumina PE500 platform. The archaea and bacteria constituted the dominant kingdoms; Actinobacteria, Proteobacteria, Bacteroidetes, and Firmicutes were the dominant bacterial phyla; and Euryarchaeota were the dominant archaeal phylum. The microbial communities showed significant structure divergence according to the salt concentration (saline (mean EC 22 mS/cm) and hypersaline (mean EC 70 mS/cm)), wherein the communities were dominated by bacteria in saline soils and archaea in hypersaline soils. Most of the dominant bacterial representation decreased with salinity, while the archaea increased with salinity. KEGG functional annotation showed that at level 2, the cell motility, environmental adaptation, signal transduction, signaling molecules and interaction, glycan biosynthesis and metabolism, and metabolism of other amino acids were reduced from saline to hypersaline, whereas the metabolism of cofactors and vitamins, folding sorting and degradation, replication and repair, transcription and translation, amino acid biosynthesis, glycolysis/gluconeogenesis, and carbon fixation increased with salinity. The increased salt content decreased the carbohydrate activities of microorganisms. The osmolyte regulation substance synthesis and absorption-related genes were more abundant in saline soils than in hypersaline soils, whereas the Na^+^/H^+^ antiporter genes (*mnhB-E*) and H^+^/Na^+^-transporting ATPase genes (*atpA-F*, *I*, *K*) were significantly higher in hypersaline soils. This indicated that in saline soils, microorganisms primarily synthesize and/or uptake compatible solutes to cope with osmotic stress, whereas in the hypersaline habitat, the high-salt-in strategy was predicated to be adopted by the halophilic/extremely halophilic microorganisms, coupled with a high abundance of replication and repair, cofactors and vitamin metabolism, nucleotide metabolism, and carbon fixation to provide energy and ensure cell regeneration. In conclusion, increases in salinity influence the microbial communities’ structure and function, as well as the adaptation of microorganisms.

## 1. Introduction

Salinization is one of the most widespread soil-degradation processes. A high concentration of salt in soil changes the availability of water and nutrients for both plants and microorganisms, reduces the species richness [1], and influences the microbial community assembly as well as diversity [2,3] and function such as microbial decomposition processes [4,5], organic carbon decomposition, and the biogeochemical cycling of nitrogen [6,7].

Many studies have demonstrated that salt is one of the most important environmental factors governing the distribution, abundance, and diversity of microorganisms [2,8,9,10], leading to the selection of specific microbiota capable of tolerating and surviving salinity stress [11]. Ma and Gong [12] recently reported that approximately 50% of the archaeal diversity and less than 25% of the total bacterial diversity has been recorded from saline soil habitats. The global microbial composition in saline soil has been demonstrated to be influenced more by salinity than by any other extreme chemical factor, such as the temperature, pH, or geographical distance [5,8,12,13].

Microorganisms in these saline or hypersaline habitats play vital roles in the global recycling of nutrients in saline environments and the biogeochemical cycles of ecosystems [12]. The functional gene diversity and composition could provide a deeper understanding of saline-soil microorganism adaptation to soil salinization as well to other stress factors. Metagenomic data found that soil increases in salinity correlated with decreases in carbohydrate metabolism and gene abundances of glycosyl transferases and glycoside hydrolases. In contrast, the enzyme-active genes of carbohydrate esterases and auxiliary activities were positively related to soil salinity [14]. The abundance of most functional genes involved in carbon degradation and nitrogen cycling correlated negatively with salinity. The salinity filtering effect shapes the soil bacterial community composition, and salinity acts as a critical inhibitor in the biogeochemical processes in estuary ecosystems [15].

Microorganisms in saline or hypersaline habitats are commonly salt tolerant (halotolerant) or halophilic, which developed multiple adaptation strategies to salt stress, either by increasing the synthesis of a variety of compatible solutes or by controlling the flux of ions across cell membranes to adjust their cell turgor [16,17,18]. The genome of halophilic archaea *Halococcus hamelinensis* revealed that putative genes/pathways involved in osmoprotection, oxidative-stress response, and UV-damage repair confer its survival in extreme salt environments [19]. The genome of nanohaloarchaeon *Candidatus* Nanopetramus SG9 revealed a photoheterotrophic lifestyle and a low median isoelectric point (pI) for all predicted proteins, suggesting a ‘salt-in’ strategy for osmotic balance [20]. The transcriptional response indicates that many genes encode for proteins with a significantly higher proportion of acidic amino acid residues upregulated to help halophilic *Eurotium rubrum* actively counteract the salinity stress, which was also shared by other halophilic prokaryotes, supporting the theory of convergent evolution under extreme hypersaline stress [21]. 

The investigation of the saline-soil microbial community composition could provide insights into the adaptation of microbiomes and plants as well as microbiome–plant interactions in salt environments. Many studies have focused on the isolation and characterization of halotolerant or halophilic bacteria and archaea from saline soils [22,23]. Until now, many salt-tolerant beneficial microorganisms, especially plant-growth-promoting rhizobacteria (PGPR), endophytes, and arbuscular mycorrhizal fungi, have been isolated from salinized soils or roots and rhizospheres associated with halophytic plants. These salt-tolerant beneficial microbes could alleviate the negative effects of salinity and improve the growth of plants by various mechanisms; for instance, members from genera *Pseudomonas*, *Bacillus*, *Exiguobacterium*, *Enterobacter*, *Lysinibacillus*, *Stenotrophomonas*, *Microbacterium*, and *Achromobacter* improved the growth and biomass production of rice plants subjected to salinity stress (see the review of [24] and the references therein). 

In desert regions, due to the dry climate, low precipitation, and strong evaporation, salts are sucked up into the soil’s upper levels, leading to soil salinization and alterations to the microbial community composition and function. A number of studies have addressed the effect of salinity on the microbial community composition and diversity, for instance, estuaries [25,26,27], solar saltern ponds [2,11], the sediments of salt lakes or sea [28,29,30,31], sea [32], and saline soils [33]. Studies regarding the function of gene diversity, composition, and driving factors, especially in arid desert areas, are relatively lacking; however, it has been posited that microbiomes show potential for biotechnological applications in the improvement and conservation of saline soils [33] and microbiome-based agriculture. Therefore, more extensive studies on the structure, diversity, and functionality of microorganisms occurring in natural saline soils are needed. In this study, we collected saline soils with different salt concentrations (EC values ranging from approximately 12 to 110 mS/cm) from an arid desert region in Xinjiang, China, sequenced and analyzed the microbial community, aiming to (1) reveal the prokaryotic community composition, structure, diversity, and function; (2) investigate the relationships between the salinity and microbial communities structure and functions, and the structural and functional difference between two salt concentrations (saline and hypersaline); and (3) discuss the salt adaptations of microorganisms to salt stress.

## 2. Materials and Methods

### 2.1. Field Investigation and Saline Soil Collection

The study area of Xinjiang is located in the northwest of China, where the climate is arid, with low precipitation and strong evaporation. Due to its distinct climate, large areas of land in this region are highly salinized. In this arid saline region, halophytic species are the dominant plants [34]. A total of 42 samples were collected from 14 sites across Xinjiang in September 2021. There was no rainfall in the recent three days at least ahead of the sampling day in each site. The geographic information of sampling sites is listed in Table S1. At each site, three samples were collected from the 0–20 cm surface layer and packed in a 50 mL sterile tube after the removal of any stones or plant debris. The samples were temporarily kept at −10 °C in a refrigerator during transportation. After returning to the laboratory, each of the samples was divided into two parts, of which one part was air-dried at room temperature for physicochemical property tests after being sieved in 2 mm mesh and the other was kept at −80 °C for genomic DNA extraction. 

### 2.2. Soil Properties Determination

The soil moisture was calculated from the weight difference of soil weighed before and after drying at 105 °C for 48 h. The soil pH was determined using an electrode pH meter (DDSJ-319L, Shanghai, China) in a 1:2.5 soil/water (*w/v*) suspension. The total organic carbon (TOC) was estimated using a UV–visible spectrophotometer (UV-1200, Shanghai, China), after soil samples were oxidized with K_2_Cr_2_O_4_. The concentration of total phosphorus (TP) and available phosphorus (AP) was determined by Mo-Sb colorimetric analysis with the UV-1200 spectrophotometer after soil was digested with a HClO_4_-H_2_SO_4_ solution for 60 min. The total nitrogen (TN) was analyzed by an AA3 flow analyzer (SEAL Analytical GmbH, Norderstedt, Germany) after digestion with concentrated sulfuric acid and perchloric acid. The nitrate–nitrogen (NO_3_^−^-N) and ammonium–nitrogen (NH_4_^+^-N) were determined by the AA3 flow analyzer after extraction in 1 mol/L KCl.

### 2.3. Soil Genomic DNA Extraction, Library Construction, and Metagenomic Sequencing

The total DNA was extracted from each soil sample (0.5 g) using a FastDNA SPIN Kit for Soil (MP Biomedicals, Cleveland, OH, USA). The quantity and quality of isolated DNA were evaluated using a NanoDrop 2000 spectrophotometer (Thermo Fisher Scientific, Waltham, MA, USA) and 1% agarose gel electrophoresis, respectively. The genomic DNA was randomly sheared into short fragments of about 350 bp using a Covaris M220 (Covaris, Woburn, MA, USA). The obtained fragments were end-repaired, A-tailed, and ligated with an Illumina adapter, then PCR amplified, size selected, and purified. The paired-end library was prepared using an Ultra DNA Library Prep Kit for Illumina (New England Biolabs, Beverly, MA, USA) and checked with a Nanodrop 2000 and real-time PCR for quantification, and the Agilent 2100 Bioanalyzer (Agilent, Santa Clara, CA, USA) was used to ascertain the size of the library. The quantified libraries were pooled and sequenced using an Illumina HiSeq PE500 platform at Novogene Bioinformatics Technology Co., Ltd. (Tianjin, China).

### 2.4. Metagenome Assembly and Function Annotation

The raw sequencing data were filtered with Readfq [35] to obtain clean data by removing reads with low-quality bases (quality threshold < 38) exceeding 40 bp length, reads with N bases reaching 10 bp, and reads overlapping with adapters >15 bp. The MEGAHIT software (v1.0.4-beta) was used to assemble the clean reads to scaffolds. The assembled Scaftigs were interrupted from the N connection, and Scaftigs without N remained [36,37].

MetaGeneMark (V3.05) [38] was used to perform ORF prediction for Scaftigs (≥500 bp) with the default parameters [39,40,41]. The CD-HIT software V4.5.8 was used to eliminate redundancy and obtain the non-redundant initial gene catalogue [42,43]. Then, the clean reads were mapped to the initial gene catalogue by using Bowtie2 [44] with ≥95% identity, and the gene abundances were evaluated in each sample. Genes with total reads ≤2 [45] in each sample were removed to acquire the final unigenes. 

The DIAMOND software (V0.9.9) [46] was used for alignment of unigenes with those of bacteria, fungi, archaea, and viruses extracted from NCBI’s NR database (Version 2018-01-02) with a cutoff e-value of 10^−5^. The LCA algorithm was adopted to determine the species annotation information by the MEGAN software [47]. The abundance at each taxonomy and the corresponding gene abundance were finally generated.

The DIAMOND software (v0.9.9) [46] was used to align unigenes with those in the functional database with a cut-off e-value of 10^−5^. The functional databases include the KEGG database [48,49], eggNOG database [50], and CAZy database [51]. The best Blast Hit results were selected for subsequent analysis. The relative abundance of COG, KEGG pathway annotation, and carbohydrate active enzyme were calculated.

### 2.5. Statistical Analysis

R software and GraphPad Prism 5 were used to conduct statistical analyses and to plot the taxonomic information at different taxonomic levels. The diversity indices were calculated using the vegan package. Principal coordinate analysis was conducted based on the Bray–Curtis distance to reveal the community clustering. PERMANOVA analysis was used to assess the similarities and significance between soil groups. General variation analysis to compare the two soil groups was conducted using the unpaired *t*-test for soil properties, and the Wilcoxon rank sum test was used to identify the abundance of taxonomy and functional categories. The false-discovery rate (FDR) was used to correct the *p*-value. Redundancy analysis (RDA) and linear regression were used to quantify the relationship between the microbial community and soil parameters, which were implemented in the R software and GraphPad Prism 5, respectively.

## 3. Results

### 3.1. Soil Characteristics

The World Reference Base for Soil Resources [52] considers the reference value 15 mS/cm of EC in defining the salic horizon, while the USDA Soil Taxonomy [53] fixed the threshold at 30 mS/cm for saline soil. The physicochemical parameters showed that the EC values of the soil ranged from 12.87 mS/cm to 110.9 mS/cm, and the pH values ranged from 7.59 to 9.76 (Table 1). We therefore divided soil samples into two groups, saline (EC < 30 mS/cm) and hypersaline (EC ≥ 30 mS/cm) soils, according to the USDA Soil Taxonomy criterion. The saline group included 18 samples from 6 sites with mean EC value of 22.42 mS/cm; the hypersaline group included 24 samples from 8 sites with mean EC value of 70.53 mS/cm. The salt content between the two groups was significant (*p* < 0.001). Apart from saline-alkaline evidenced by the EC and pH, the studied soils were also nutrient poor, with average SOC, TN, TP, NO_3_^−^-N, and NH_4_^+^-N contents of 7.65 ± 1.05 g/kg, 0.62 ± 0.11 g/kg, 0.47 ± 0.02 g/kg, 0.133 ± 0.020 g/kg, and 2.06 ± 0.57 mg/kg, respectively. In addition, the contents of AP, SOC, TN, and NH_4_^+^-N in saline soils were significantly higher than those in hypersaline soils (*p* < 0.05). The soil moisture contents ranged from 1.34% ± 0.17% to 22.32% ± 0.84%, with a mean of 7.85% ± 5.16%. In general, the saline and hypersaline soil differed significantly in terms of most of the geochemical parameters (*p* < 0.05), and the increased salt content reduced the soil nutrient quality. 

### 3.2. Sequencing Data and Metagenome Assembly

From the metagenomic sequencing, 684.89 Gbp of raw reads were obtained from 42 samples, with an average of 16.31 Gbp per sample. After quality control, a total of 680.11 Gbp clean reads was retained with an average of 16.193 Gbp of each sample. The clean reads were assembled into 581,782 contigs (>500 bp), consisting of 23.135 Gbp, and a GC content of 64.09%. The detailed sequencing data and metagenome assembly statistics in each sample are listed in Table S2. 

A total of 22,237,054 unigenes were identified, with a total gene length of 12,239.5 Mbp and average gene length of 550.41 bp. A total of 75.11% of the total genes were annotated on NR, and 14.15% were annotated as unclassified. A total of 85.85%, 80.34%, 77.34%, 67.82%, 64.72%, 57.27%, and 45.16% were annotated on kingdom, phylum, class, order, family, genus, and species level, respectively. A total of 46% were affiliated to bacteria, 28.34% were identified as archaea, 0.059% belonged to Eukaryota, and viruses accounted for 0.25%. At the phylum level, the unigenes were mainly assigned into Euryarchaeota (accounting for 36.40% of the total unigenes), Actinobacteria (16.29%), Proteobacteria (9.64%), Bacteroidetes (8.7%), Firmicutes (2.16%), Gemmatimonadetes (1.21%), Balneolaeota (1.25%), and Chloroflexi (1.19%). At the genus level, the most abundant genera were *Nitriliruptor* (3.84%), *Halalkalicoccus* (3.12%), *Natronomonas* (2.87%), *Staphylococcus* (0.71%), *Gillisia* (1.71%), *Lawsonella* (0.19%), *Prevotella* (0.26%), *Halosimplex* (0.83%), *Salinimicrobium* (0.71%), and *Gemmatimonas* (0.69%) (Table S3). 

### 3.3. Microbial Community Composition and Structure

**Figure 1:**
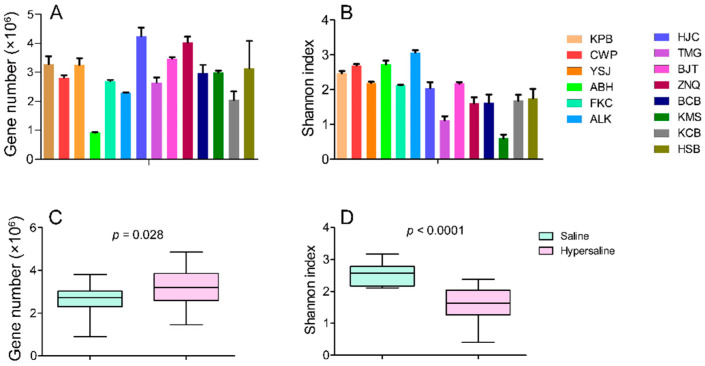
Gene numbers (**A**) and Shannon diversity (at phylum level) (**B**) of microbial communities in each site; and difference in gene number (**C**) and Shannon diversity (**D**) between saline and hypersaline soils.

Gene numbers ranged from 921,921 to 4,242,615 in each site. The Shannon index ranged from 5.04 ± 0.10 to 7.51 ± 0.26 with a mean of 6.32 ± 0.70 at the genus level, and from 0.61 ± 0.18 to 3.07 ± 0.11 with an average of 1.99 ± 0.67 at the phylum level (Figure 1, Table S4). 

**Figure 2:**
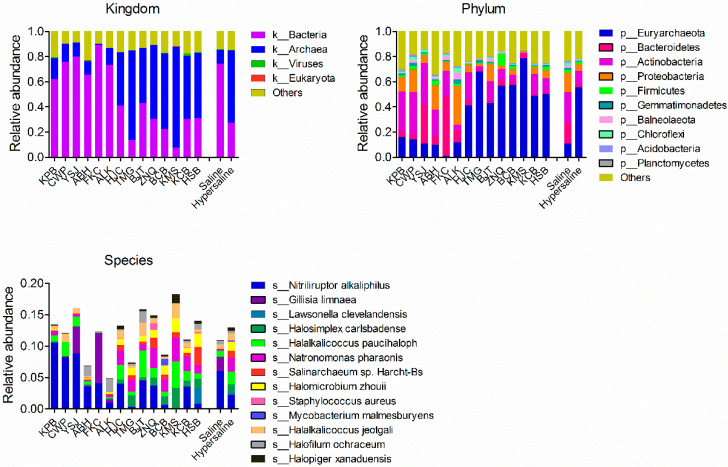
Microbial community composition at the kingdom, phylum, and species level in each site and soil group. Saline and hypersaline represent combined saline soils and hypersaline soils, respectively.

The microbial communities were dominated by bacteria and archaea. The most abundant phyla were Euryarchaeota, Actinobacteria, Proteobacteria, Bacteroidetes, Firmicutes, and Gemmatimonadetes. The most abundant species were *Nitriliruptor alkaliphilus*, *Gillisia limnaea*, *Lawsonella clevelandensis*, *Halosimplex carlsbadense*, *Halalkalicoccus paucihalophilus*, *Natronomonas haraonic*, *Salinarchaeum sp*. Harcht-Bsk1s, *Halomicrobium zhouii*, *Staphylococcus aureus*, and *Mycobacterium malmesburyense* (Figure 2). 

**Figure 3:**
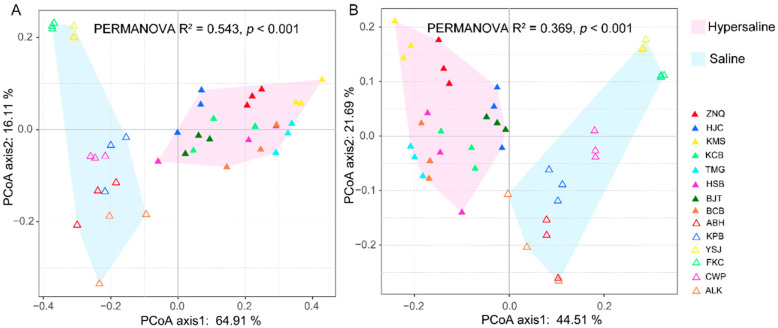
PCoA plot at the phylum (**A**) and genus (**B**) level illustrating the separation of microbial communities along axis 1 corresponding to the saline and hypersaline soils.

**Figure 4:**
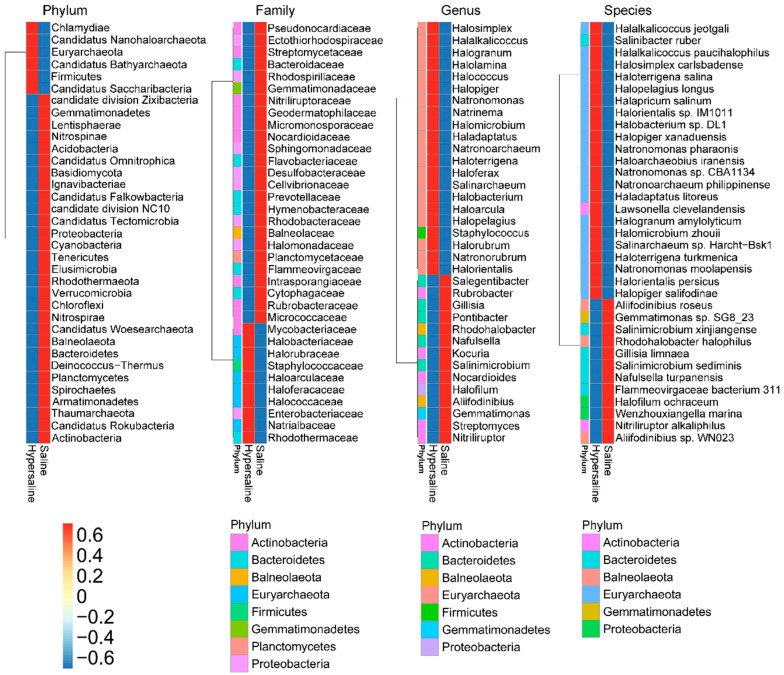
Difference analysis of the most abundant taxonomic groups between saline and hypersaline soils at phylum, family, genus, and species level.

The microbial communities structure showed a significant difference between the saline and hypersaline soils that they were clearly separated along axis 1 in PcoA plot (PERMANOVA R^2^ = 0.543, *p* < 0.001 at phylum level, and R^2^ = 0.369, *p* < 0.001 at genus level) (Figure 3). The abundance of archaea was significantly higher in hypersaline soils than in saline soils, whereas for the bacteria it was the opposite (*p* < 0.001). The saline soils were dominated by bacteria (accounting for 74.3% of total genes), whereas the archaea represented the dominant kingdom (accounting for 57.3% of total genes) in hypersaline soils (Figure 2, Figure 4). In the saline group, the archaea had a significantly lower gene abundance than bacteria (404,100 ± 65,820 vs. 1,866,000 ± 164,800, *p* < 0.001), whereas in the hypersaline soils, the gene abundance of archaea was significantly higher than bacteria (2,055,000 ± 107,600 vs. 797,900 ± 99,890, *p* < 0.001). A similar pattern was also observed at a lower taxonomic level. The relative abundances of the most abundant bacterial phyla, Bacteroidetes, Actinobacteria, and Proteobacteria, were significantly higher in saline soils than in hypersaline soils, while the Euryarchaeota abundance was lower (Figure 4). The relative abundances of genera *Gillisia*, *Nitriliruptor*, *Halalkalicoccus*, *Natronomonas*, *Prevotella*, and *Salinimicrobium* were higher in saline soils than in the hypersaline soils; in contrast, the relative abundances of the dominant genera in hypersaline soils were approximately 10 times more than in saline soils, such as *Haladaptatus*, *Haloterrigena*, *Halomicrobium*, *Halorientalis*, *Halococcus*, *Halorubrum*, *Halopiger*, *Halosimplex*, *Halobacterium*, *Natrinema*, and *Staphylococcus* (Table S5). 

The microbial community diversity was significantly higher in saline soils than in hypersaline soils at both the phylum and genus level (Figure 1, Table S4). This indicated that the salinity was an important factor in shaping the microbial community, in which high salt caused the community structure to change from bacteria to archaea. Indeed, studies have already indicated that archaea are more adaptive to extreme environments than bacteria [19,20]. 

Notably, we also found a significant difference in microbial community structure between two sites (FKC and YSJ) and other sites within saline groups. The PCoA plot showed that FKC and YSJ were clustered together and separated with other saline samples along axis 2 (Figure 3). FKC and YSJ predominantly contained phyla Bacteroidetes and a low abundance of Proteobacteria and Euryarchaeota, while other sites had a high abundance of Proteobacteria and Euryarchaeota and low abundance of Bacteroidetes (Figure 2). 

### 3.4. Function Genes Composition

Here, 60.06% of the total unigenes were annotated on eggNOG, 63.85% on KEGG, 31.66% on KO, and 2.95% on CAZy. 

**Figure 5:**
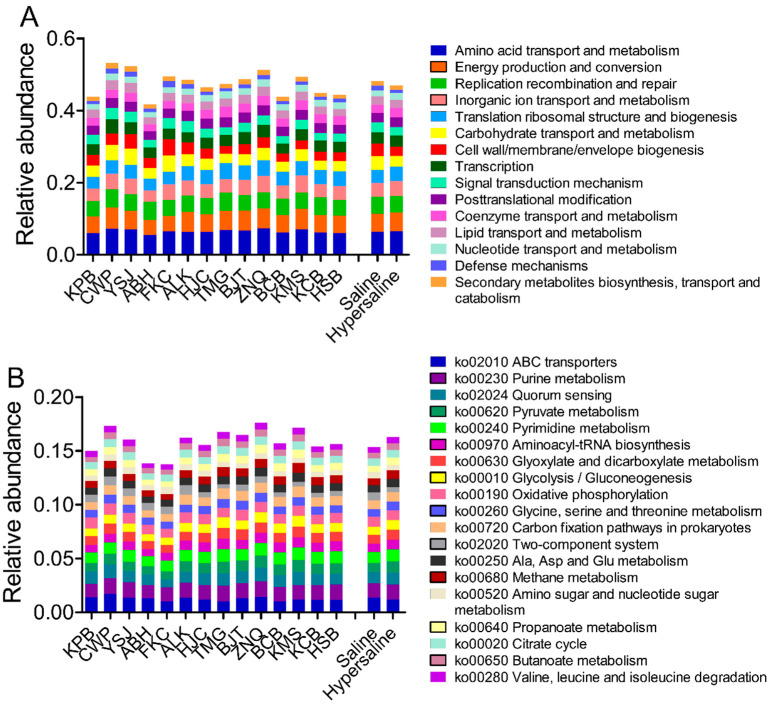
Relative abundance of genes annotated in COG at level 1 (**A**) and KEGG at level 3 (**B**) of microbial communities in each site, and combined saline and hypersaline soils.

Clusters of orthologous groups of proteins (COG) analysis revealed that 5.06% of unigenes hit in energy production and conversion; 6.53% unigenes annotated on amino acid transport and metabolism; 4.62% on replication, recombination, and repair; 3.97% on inorganic ion transport and metabolism; 3.32% on carbohydrate transport and metabolism; 3.91% on translation, ribosomal structure, and biogenesis; 3.12% on transcription; and 1.09% of the genes were involved in defense mechanisms (Figure 5, Table S6). The translation, ribosomal structure, and biogenesis, RNA processing and modification, coenzyme transport and metabolism, nucleotide transport, and metabolism were more abundant in hypersaline soils, whereas the defense mechanisms, cell motility, carbohydrate transport and metabolism, cell wall/membrane/envelope biogenesis, and signal transduction mechanisms were, in contrast, less abundant (Figure S1).

KEGG functional classification showed that genes were associated with metabolism, genetic information processing, environmental information processing, and cellular processing at level 1 (Figure S2). In metabolism, 2.78% of genes were involved in energy metabolism, 4.26% in carbohydrate metabolism, 0.54% in glycan biosynthesis and metabolism, 4.25% in amino acid metabolism, 1.02% in the metabolism of other amino acids, 2.67% in the metabolism of cofactors and vitamins, 1.71% in nucleotide metabolism, 1.09% in lipid metabolism, and 0.83% in the biosynthesis of other secondary metabolites at level 2. There was 0.05% of genes involved into environmental adaptation.

In total, 419 KEGG pathways were annotated at level 3. The most abundant pathways were associated with ABC transporters, nucleotide metabolism, carbohydrate metabolism (pyruvate, glycolysis/gluconeogenesis, carbon fixation, citrate cycle, pentose phosphate pathway), multiple kinds of amino acid metabolism, and oxidative phosphorylation. The DNA replication, nitrogen metabolism, and fatty acid biosynthesis also had relative high abundances (Figure 5). 

**Figure 6:**
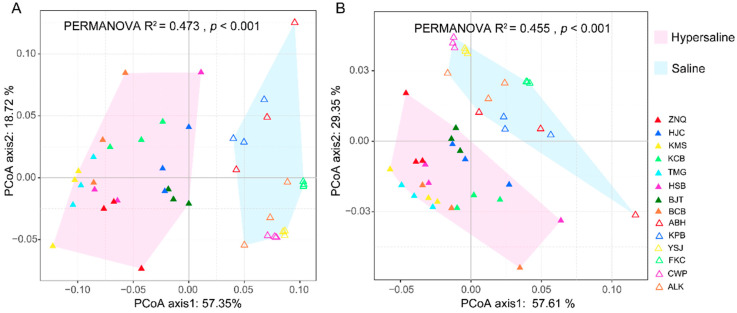
PCoA plot indicating that the saline and hypersaline microbial communities were separated at KEGG orthologues (KO) (**A**) and KEGG pathway level 3 (**B**).

PCoA based on the functional composition showed that two separate clusters were formed corresponding to the salinity gradient (Figure 6), as illustrated in microbial composition (Figure 3), indicating that salinization also led to significant differences in the soil microbial functions. 

**Figure 7:**
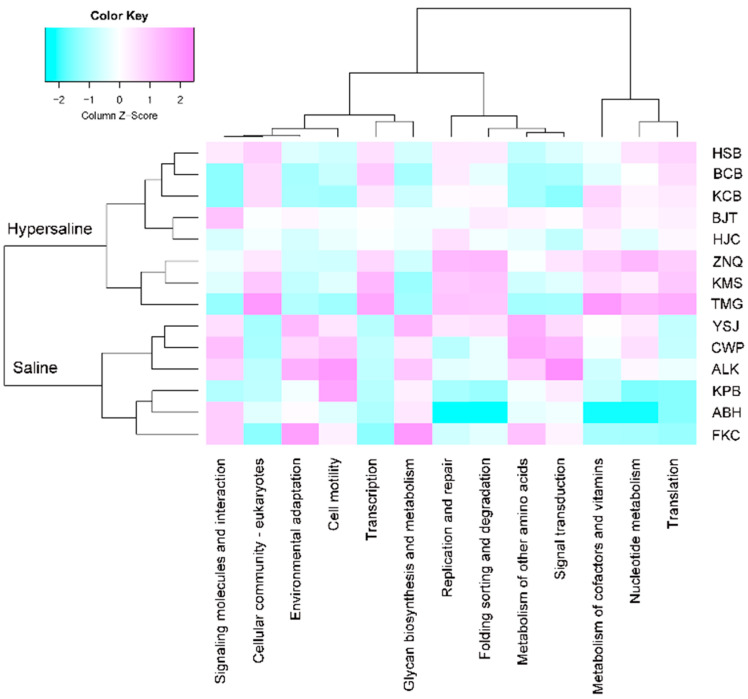
Heatmap of different KEGG pathways at level 2 between the saline and hypersaline soils (FDR < 0.05).

Differential analysis revealed that the metabolism of cofactors and vitamins, nucleotide metabolism, and membrane transport were significantly higher in hypersaline soils than in saline soils (Figure S3). At level 2, the cell motility, environmental adaptation, signal transduction, signaling molecules and interaction, glycan biosynthesis and metabolism, and metabolism of other amino acids were reduced from saline to hypersaline, whereas xenobiotics biodegradation and metabolism, metabolism of cofactors and vitamins, folding sorting and degradation, replication and repair, transcription, and translation increased with salinity (Figure 7). At KEGG level 3, 129 pathways were significantly different between the two groups, of which 65 were more abundant in the hypersaline group, and the remaining 64 were more enriched in saline group, including some pathways in amino acid metabolism (i.e., metabolism of alanine, aspartate and glutamate, glycine, serine and threonine, and histidine, biosynthesis of arginine, lysine, phenylalanine, tyrosine and tryptophan, valine, leucine and isoleucine), carbohydrate metabolism (i.e., ascorbate and aldarate, glyoxylate and dicarboxylate, pyruvate, glycolysis/gluconeogenesis), carbon fixation, and methane metabolism (Table S7).

**Figure 8:**
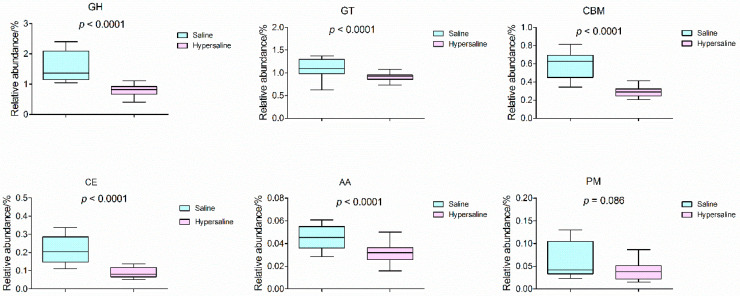
The differential analysis of relative abundance for carbohydrate-active enzymes between saline and hypersaline soils.

CAZy analysis showed that the most abundant enzymes were glycoside hydrolases (GHs), glycosyl transferases (GTs), and carbohydrate-binding modules (CBMs), whereas the carbohydrate esterases (CEs), polysaccharide lyases (PLs), and auxiliary activities (AAs) were less abundant. All of the carbohydrate active enzymes in hypersaline soils were significantly lower than in saline soils, except for the PM (Figure 8), indicating that the increased salt content decreased the carbohydrate activities of microorganisms.

### 3.5. Driving Factors and Contributions for Community Structure and Function

**Figure 9:**
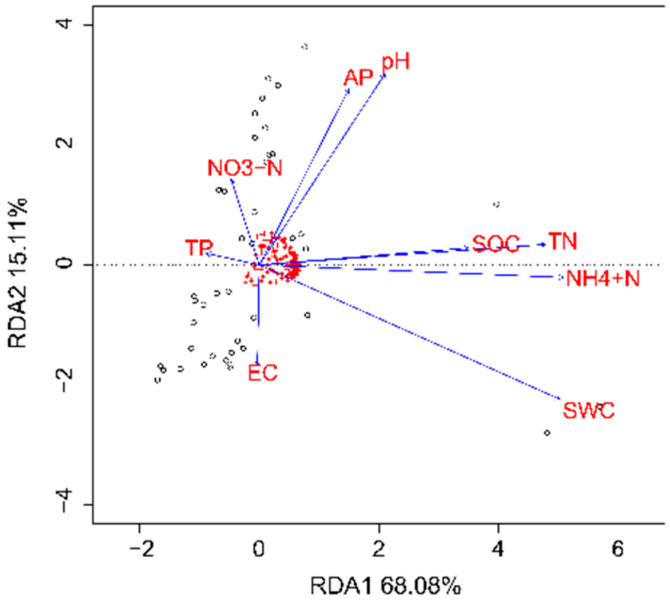
The redundancy analysis (RDA) illustrating the correlations between soil properties and microbial structure at the level of phyla.

**Figure 10:**
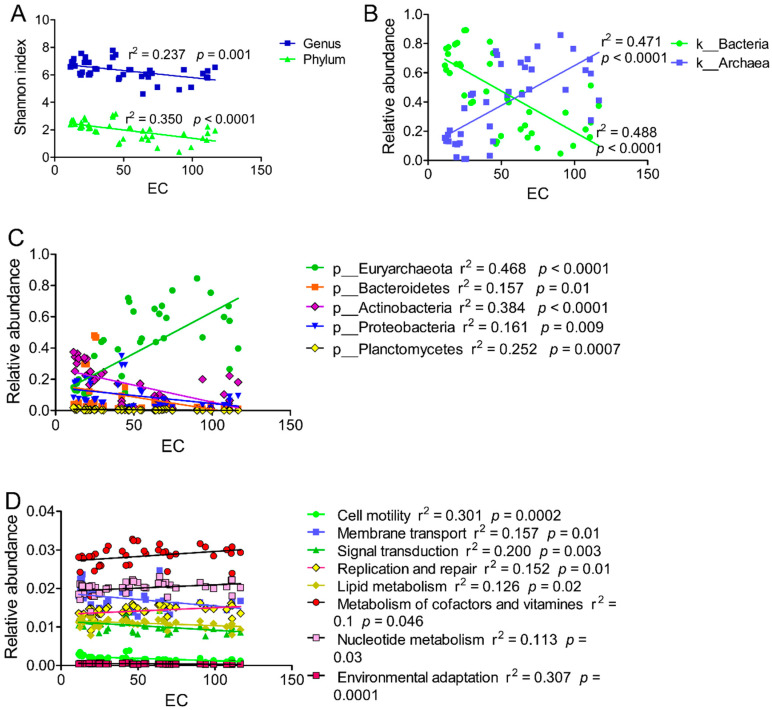
Regression analysis of EC and Shannon index (**A**), and relative abundance at kingdom (**B**) and phylum level (**C**), and KEGG at level 2 (**D**).

RDA showed that the first two axes could explain 83.17% of the total community variance (Figure 9). Linear regression analysis showed that the EC, pH, AP, and TN were significantly correlated with the abundance of bacteria and archaea (Table 2). The presence of archaea was positively correlated with EC and negatively correlated with pH, AP, and TN; in contrast, the presence of bacteria was negatively correlated with EC and positively correlated with pH, AP, and TN. EC was the first influential factor shaping the microbial community structure and diversity, and it was significantly negatively correlated with microbial community diversity and significantly positively correlated with the abundance of archaea, but negatively with bacteria (Figure 10A,B). At a lower taxonomic level, EC was significantly positively correlated with abundance of Euryarchaeota but negatively correlated with abundance of Bacteroidetes, Actinobacteria, proteobacteria, and Planctomycetes (Figure 10C).

At KEGG level 2, linear regression revealed that the EC was positively correlated with replication and repair, metabolism of cofactors and vitamins, and nucleotide metabolism, and negatively correlated with membrane transport, environmental adaptation, cell motility, signal transduction, and lipid metabolism (Figure 10D).

Among the 419 level 3 pathways, 133 were significantly correlated with EC, of which 34 were positively correlated and 99 were negatively correlated (Table S8). With a salt increase, the abundance of genes decreased related to pathways, including metabolism of fructose and mannose, starch and sucrose, sulfur, arachidonic acid, and linoleic acid, biosynthesis of fatty acid, unsaturated fatty acid, secondary metabolite (such as betalain, flavonoid, indole alkaloid, and isoflavonoid), biosynthesis and degradation of several terpenoids and polyketides, and photosynthesis. However, only fewer pathways positively related to EC, for instance, cell cycle, DNA replication, RNA transport, proteasome, basal transcription factors, phenylalanine, biosynthesis of tyrosine and tryptophan, isoquinoline alkaloid, tropane, piperidine and pyridine alkaloid, and N-Glycan. Overall, as indicated by its higher correlation coefficient, soil salinity was the strongest influencing factor among the examined variables.

### 3.6. Salt Adaptation Related Genes and Mechanism

**Figure 11:**
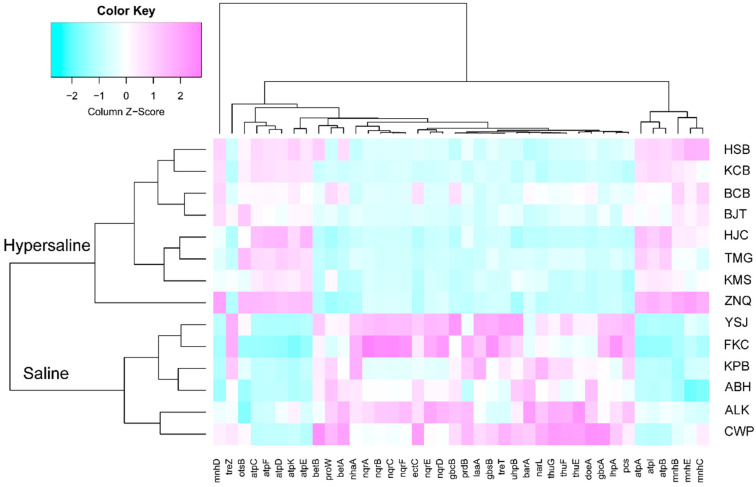
Heatmap of significantly different genes (FDR < 0.05) between the saline and hypersaline soils involved in compatible organic osmolytes synthesis and metabolism and ions flux controlling channel aquaporin.

In this study, genes involved in compatible osmolytes synthesis, i.e., betaine, choline, ectoine, proline, and trehalose, and ions flux controlling channel aquaporin, i.e., cation, cation/H, and H/Na, were selected and compared on their presence in communities of two types of soils. The glycine betaine catabolism can contribute to microbiome tolerance to hyperosmotic stress [54]. Betaine is a precursor of choline, phosphocholine, and glycine. The genes related with betaine (*gbcA*, *gbcB*, *betB*), choline (*betA*, *gbsB*, *pcs*), ectoine (*doeA*, *ectC*), proline (*laaA*, *prdB*, *lhpA*), trehalose (*treT*, *thuG*, *sugB*, *thuF*, *sugA*, *thuE*, *treZ*, *glgZ*) synthesis were significantly higher in saline soils than in hypersaline soils (FDR < 0.05). The Na^+^-translocating NADH: quinone oxidoreductase (Na^+^-NQR) is a unique prokaryotic respiratory enzyme pumping of Na^+^ across the cell membrane, which is used by the cells to transport nutrients, secrete toxins and antibiotics, and maintain ion homeostasis [55]. Na^+^-NQR coding genes *nqrA-F* were more enriched in saline soils than in hypersaline soils. In contrast, the Na^+^/H^+^ antiporter genes (*mnhB-E*) and V/A-type Na^+^/H^+^-transporting ATPase genes (*atpA-F*, *I*, *K*) were significantly higher represented in hypersaline soils than in saline soils (Figure 11, Table S9). 

## 4. Discussion

### 4.1. Communiuty Structure

A meta-analysis of soil microbial communities along salinity gradients found a significant reduction in prokaryotic alpha-diversity indices with increasing salinity, and microbial community composition changes demonstrated that the microbial communities were influenced by soil salinity in the arid region [56] owing to the salinity selection [15,57]. This study found that the archaeal community showed a richness and diversity significantly affected by the spatial gradients of soil salinity; conversely, the bacteria showed a decreasing trend with increasing gradient of soil salinity. This is consistent with findings previously reported in salt lake sediments of Xinjiang [31], plateau lakes in the Qaidam Basin [58], and a semiarid hypersaline Mediterranean area [33]. 

The predominant prokaryotic phyla were Proteobacteria, Actinobacteria, Bacteroidetes, Firmicutes, and Euryarchaeota, consistent with findings previously reported on salt lake sediments [31]. Euryarchaeota dominate in extreme environments, saline to hypersaline soils, salt lakes and oceans, and glacial lake sediments [59,60]. Its prevalence indicates its adaptation to various harsh environments. 

The phyla Bacteroidetes, Actinobacteria, and Proteobacteria accounted for the majority of the bacterial community in saline soils, which had higher salt tolerance [15,61,62]. For example, the bacteria of the family Alteromonadaceae (Gammaproteobacteria) was observed in its highest abundance in hypersaline soils [57,63]; the highly salt-tolerant Flavobacteriaceae (Bacteroidetes), Rhodobacteraceae (Alphaproteobacteria), and Nitriliruptoraceae (Actinobacteria) were found dramatically enriched in the hypersaline plateau lakes [64]. However, significant changes in the microbial community composition due to soil salinization were observed. With salt concentration increases from 40 to 110 mS/cm, the abundance decreased significantly in hypersaline soils, wherein only a small proportion of extremely halophilic bacteria and archaea can survive. Even the above mentioned highly salt-tolerant bacteria [57,63,64] also decreased significantly in hypersaline soils in the present study with EC values of 70 mS/cm, which might be beyond their optimal tolerance thresholds. Interestingly, the Firmicutes abundance increased from the saline to hypersaline soils, perhaps because the Firmicutes harbor several species which can resist harsh environmental stresses due to their Gram-positive cell walls and spore-forming ability [65]. Li et al. [31] observed that the communities were dominated by bacteria in lakes with salinities of <100 g/L and by archaea (accounting for 79.1 to 89.4%) in Lake Gasikule where the salinity was 317 to 344 g/L. Canfora et al. [33] also reported that the microorganisms in a hypersaline environment change from moderately halophilic bacteria to communities of extremely halophilic bacteria and archaea. 

At a lower taxonomic level, the abundant genera and species were different from other studies conducted in diverse regions and habitats [31,66]. We also found that the YSJ and FCK community structures differed from other saline sites. This indicates that other factors, i.e., geography, pH, water, and nutrients also play important roles in structuring the microbial community [15], as suggested by the linear regression analysis.

### 4.2. Function Composition

Prokaryotes in nutrient-poor and devoid of vegetation, highly saline, and alkaline environments are ecologically important microorganisms that are responsible for the decomposition, mineralization, and subsequent recycling of organic matter [12,56,67]. We found that the abundances of genes associated with carbohydrate metabolism, amino acid metabolism, and energy metabolism were not significantly affected by the salt concentration. This implies that the basic nutrient and energy activities of essential microbiomes are independent of the salt concentration. Further, when salt stress is increased, the genes involved in translation, transcription, replication and repair, metabolism of cofactors and vitamins, and nucleotide metabolism are much higher. Probably, high salt stress damages the structural molecules and function and stimulates cell regeneration. 

However, there was significant difference between the two saline gradients regarding the abundance of some pathways (level 3) in carbohydrate, amino acid, energy, and lipid metabolism. For instance, the carbon fixation and methane metabolism pathway in the energy metabolism increased with salinity; in contrast, the sulfur metabolism and photosynthesis decreased with salinity. Similarly, for the carbohydrate metabolism, the gene abundance involved in starch and sucrose, fructose and mannose, inositol phosphate, and amino sugar and nucleotide sugar metabolism pathway were much higher in saline soils, while gene related with ascorbate and aldarate metabolism, glycolysis/gluconeogenesis, pyruvate, and glyoxylate and dicarboxylate metabolism were much higher in hypersaline soils (Table S8). These results imply that, under different salt stress conditions, microorganisms positively regulate cell metabolism to maintain survival by maintaining the stability of some key pathways and downregulating or upregulating other pathways. However, some key carbohydrate and energy metabolism pathway (i.e., citrate cycle, pentose phosphate pathway, nitrogen metabolism, and oxidative phosphorylation) were not significantly different.

### 4.3. Driving Factors and Contributions to Community Structure and Function

At a global scale, salinity has been demonstrated to be one of the essential factors affecting microbial distribution, microbial communities’ biological structure and functions in soil environments [68]. This study found that the overall microbiome abundance decreases with a salinity increase, and the community converts from bacteria dominance to archaea dominance as the bacteria abundance decreases but archaea abundance increases with a salinity increase. We observed that a large part of bacterial genera decreased in abundance; in particular, some beneficial microbiomes decreased in abundance, such as *Streptomyces*, *Azospirillum*, *Bacillus*, *Pseudomonas*, *Serratia*, *Klebsiella*, *Arthrobacter*, *Paenibacillus*, *Kocuria*, and *Halomonas*. Many members of these genera are salt-tolerant PGPR that can enhance plant salt tolerance and improve growth and performance (see review of [24] and the references therein). The reduction in abundance of these PGPRs might be unfavorable for plant growth and agricultural productivity. 

With soil change from saline to hypersaline, some of the pathways are positively related to EC, such as replication and repair, cofactors and vitamin metabolism, and nucleotide metabolism. At a lower pathway level, a large number of pathways represented negative correlations with salinity, such as fructose and mannose metabolism, unsaturated fatty acid biosynthesis, and secondary metabolite biosynthesis (i.e., betalain, flavonoid, indole alkaloid, and isoflavonoid). In contrast, some pathways showed an opposite trend, including DNA replication, RNA transport, proteasome, cell cycle (Table S8). Further, the carbohydrate active enzyme activities also decreased significantly. Overall, the soil salinity acts as a critical impact factor for soil biogeochemical processes [15].

The increase in salinity significantly reduces the soil microbial community diversity structure and function [2,66], which suggests that soil salinization poses a negative effect on ecosystem health. Xinjiang is located at the interior of northwest China, where the arid climate means that there is high soil salinization across large land areas, raising multiple ecological problems, such as the reduction in soil quality and vegetation coverage and loss of agricultural productivity. Thus, the prevention of soil salinization and improvement of saline alkaline soil is urgent. The maintenance of the high abundance of salt-tolerant beneficial microorganisms in soils has great potential in increasing stress resistance, promoting growth, and enhancing the biomass and productivity of agricultural plants in salinized soils [69]. Therefore, it is essential to reduce the salt concentration of the hypersaline soils for improvement of the soil microbial community structure and enhancement of the soil function. 

### 4.4. Salt Adaptation Related Genes and Mechanism

Microorganisms in saline or hypersaline habitats are usually salt tolerant (halotolerant) or halophilic, and have developed multiple adaptations to salt stress. For instance, the ‘high-salt-in strategy’ microbes accumulate the salt ions to balance the cytoplasmic osmotic pressure [16], and the ‘organic-solutes-in strategy’ microbial species synthesize and/or uptake low-molecular-weight organic compatible molecules to exclude salt ions from the cytoplasm to cope with osmotic stress [17]. 

This study found that Actinobacteria, Bacteroidetes, Gammaproteobacteria, and Alphaproteobacteria were more abundant in saline soils; many members affiliated to these phyla are salt tolerant. Correspondingly, the pathway and genes involved in osmotic substance (betaine, choline, ectoine, proline, trehalose) synthesis were higher; moreover, the Na^+^-NQR coding genes *nqrA-F* were also enriched in saline soils. These indicate that bacteria adopt the organic-solutes-in strategy by increasing the synthesis or absorption of a variety of compatible organic osmolytes or by controlling the flux of ions across cell membranes to resist salt stress. 

When the soils salt concentration increases from saline to hypersaline in the arid Xinjiang, the community composition changed from salt-tolerant bacteria to archaea, which was primarily dominated by halophilic/extremely halophilic or alkaliphilic archaea. Several families, such as Haloarculaceae, Halobacteriaceae, Halococcaceae, Halorubraceae, and Natrialbaceae, increase in abundance, which commonly require a relatively high concentration of ions for growth [70,71,72,73,74]. Therefore, they are very well-adapted to hypersaline environments and thrive in many different hypersaline areas. Meanwhile, function analysis also showed that the pathway of metabolism of cofactors and vitamins, folding sorting and degradation, replication and repair, transcription and translation, biosynthesis of amino acids (i.e., arginine, lysine, phenylalanine, tyrosine, tryptophan, valine, leucine, and isoleucine), carbohydrate metabolism (i.e., ascorbate and aldarate, glyoxylate and dicarboxylate, pyruvate, and glycolysis/gluconeogenesis), and carbon fixation increased with salinity. Moreover, Na^+^/H^+^ antiporter genes are much more abundant in hypersaline soils than in saline soils. The Na^+^/H^+^ antiporters are membrane proteins that play a major role in the pH and Na^+^ homeostasis of cells and reduce the Na^+^ toxicity [75]. These results indicated different adaption mechanisms of microorganisms in hypersaline compared to saline soils. In a high-salt environment, the microorganisms accumulate a high concentration of ions in the cell; they might regulate Na^+^ homeostasis by ion transporters. The energy needed by the ion member transport might be supplied by ascorbate and aldarate metabolism, glyoxylate and dicarboxylate metabolism, pyruvate metabolism, TCA cycle, pentose phosphate pathway, and glycolysis/gluconeogenesis. 

The saline environments are usually oligotrophic, and the hypersaline soils are even much less nutrient poor. Therefore, the autotrophic processes might be essential for microbiomes [60], as supported by the boosted carbon fixation process of microbial communities in hypersaline soils (Table S7). The accumulated high concentration of salt ions could pose stress pressure to the cellular structural substance and functions, such as proteins, enzymes, and nucleotides; therefore, microorganisms enhanced the metabolism of cofactors and vitamins, replication and repair, transcription and translation, and biosynthesis of amino acids to stimulate cell regeneration, which is supported by our findings that the cell-cycle pathway in hypersaline communities is significantly higher than in saline communities. 

## 5. Conclusions

This study investigated the microbial community structure and function as well as the relationships between the microbiome composition and function with soil salinity ranging from saline to hypersaline in arid region. As the salt concentration increased from saline to hypersaline, the bacterial abundance decreased while the archaeal abundance increased, and the microbial communities changed from bacteria dominant to archaea dominant. Salinity also affected the microbiome function: in the hypersaline environment, genes associated with environmental adaptation, some pathways of metabolism, and the carbohydrate active enzymes decreased, whereas the replication and repair, cofactors and vitamin metabolism, nucleotide metabolism, and carbon fixation were enriched. These suggest that higher salinity could influence the structure and function of the microbial community as well as the soil nutrient cycle and soil quality. Therefore, salinization should be prevented, and effective measures should be taken to improve the microbial community composition, maintain the nutrient fixation and potential salt-tolerant plant growth promotion organisms, and improve the soil nutrient cycling and quality, especially in hypersaline soils. Moreover, salinity also influences the adaptation mechanisms of the microbial community to salt stress. In the saline environment wherein bacteria were dominant, the organic-solutes-in strategy is predicated as the major mechanism to cope with osmotic stress, whereas in the hypersaline habitat, the high-salt-in strategy was mainly adopted by the halophilic/extremely halophilic microorganisms, coupled with high abundance of replication and repair, cofactors and vitamin metabolism, and nucleotide metabolism, and carbon fixation functioning to provide energy and ensure cell regeneration.

## Figures and Tables

**Table 1:** Soil physicochemical properties in each geographic site, saline and hypersaline soils.

Name	AP (mg kg^−1^)	EC (mS cm^−1^)	pH	SOC (g kg^−1^)	TN (g kg^−1^)	TP (g kg^−1^)	NO_3_^−^-N (mg kg^−1^)	NH_4_^+^-N (mg kg^−1^)	SWC (%)
ZNQ	13.40 ± 0.62	64.47 ± 0.90	8.16 ± 0.07	9.25 ± 0.24	0.49 ± 0.01	0.48 ± 0.01	156.6 ± 1.98	1.12 ± 0.01	5.59 ± 0.43
HJC	3.97 ± 0.05	28.2 ± 3.1	7.90 ± 0.00	5.68 ± 0.24	0.30 ± 0.00	0.52 ± 0.00	21.38 ± 0.63	0.83 ± 0.02	3.74 ± 0.39
KMS	10.51 ± 1.49	88.03 ± 7.17	7.74 ± 0.05	7.14 ± 0.52	0.37 ± 0.01	0.70 ± 0.01	100.90 ± 3.74	0.82 ± 0.10	2.81 ± 0.32
KCB	3.55 ± 0.25	106.00 ± 6.60	7.93 ± 0.05	6.14 ± 0.64	0.24 ± 0.00	0.46 ± 0.01	32.62 ± 0.76	0.66 ± 0.03	5.65 ± 0.40
TMG	3.83 ± 0.12	47.73 ± 1.05	7.88 ± 0.05	1.61 ± 0.26	0.20 ± 0.00	0.52 ± 0.01	162.8 ± 4.46	0.45 ± 0.08	10.89 ± 0.26
HSB	23.87 ± 0.38	110.90 ± 0.20	8.70 ± 0.01	12.68 ± 0.07	0.55 ± 0.00	0.37 ± 0.00	242.4 ± 6.36	1.74 ± 0.05	9.10 ± 0.47
BJT	5.58 ± 0.38	49.60 ± 4.91	8.39 ± 0.07	6.93 ± 0.19	0.28 ± 0.01	0.44 ± 0.01	115.4 ± 4.38	2.24 ± 0.15	5.89 ± 0.94
BCB	2.60 ± 0.81	69.27 ± 0.81	7.59 ± 0.01	3.28 ± 0.17	0.39 ± 0.01	0.41 ± 0.01	208 ± 3.32	1.45 ± 0.09	10.31 ± 1.17
ABH	4.17 ± 0.22	21.08 ± 0.90	8.22 ± 0.02	1.43 ± 0.14	0.18 ± 0.00	0.27 ± 0.00	5.64 ± 0.28	0.49 ± 0.06	11.99 ± 0.40
KPB	4.97 ± 0.97	12.87 ± 0.97	7.87 ± 0.01	2.49 ± 0.05	0.30 ± 0.01	0.47 ± 0.01	143.10 ± 3.46	1.10 ± 0.05	4.54 ± 0.53
YSJ	11.22 ± 0.34	19.07 ± 0.48	8.33 ± 0.01	2.16 ± 0.05	0.33 ± 0.00	0.49 ± 0.00	120.20 ± 2.61	1.32 ± 0.06	11.99 ± 0.40
FKC	25.44 ± 0.72	25.33 ± 0.35	8.62 ± 0.03	4.14 ± 0.37	0.33 ± 0.01	0.39 ± 0.01	16.01 ± 1.67	0.91 ± 0.05	4.54 ± 0.53
CWP	45.52 ± 3.55	13.27 ± 0.45	9.76 ± 0.02	25.28 ± 0.07	2.46 ± 0.05	0.64 ± 0.02	531.00 ± 6.93	2.53 ± 0.18	8.60 ± 0.18
ALK	14.92 ± 1.49	42.93 ± 1.27	8.65 ± 0.03	18.86 ± 0.18	2.32 ± 0.10	0.43 ± 0.01	8.51 ±0.43	13.15 ± 4.94	22.32 ± 0.84
Total Mean	12.40 ± 1.83	49.91 ± 5.08	8.27 ± 0.08	7.65 ± 1.05	0.62 ± 0.11	0.47 ± 0.02	133.2 ± 20.76	2.06 ± 0.57	7.85 ± 0.80
Hypersaline	8.41 ± 1.44 ^b^	70.53 ± 28.52 ^a^	8.04 ± 0.07 ^a^	6.59 ± 0.67 ^b^	0.35 ± 0.02 ^b^	0.49 ± 0.02 ^a^	130.05 ± 15.29 ^b^	1.16 ± 0.12 ^b^	6.74 ± 0.61 ^b^
Saline	17.71 ± 3.51 ^a^	22.42 ± 10.48 ^b^	8.58 ± 0.14 ^a^	9.06 ± 2.29 ^a^	0.98 ± 0.24 ^a^	0.45 ± 0.03 ^a^	137.41 ± 44.74 ^a^	3.25 ± 1.29 ^a^	9.32 ± 1.63 ^a^

SWC, soil water content; EC, electrical conductance; SOC, soil organic carbon content; TN, soil total nitrogen content; TP, soil total phosphorous content; AP, soil available phosphorous content; NO_3_^−^-N, soil nitrate nitrogen; NH_4_^+^-N, soil ammonium nitrogen. Different lowercase letters represents significant difference between saline and hypersaline soils at *p* < 0.05.

**Table 2:** Results of the linear regression analysis between the abundance of archaea and bacteria and soil properties.

Soil Property	Archaea				Bacteria			
	Pearson r	r^2^	F	*p*	Pearson r	r^2^	F	*p*
EC	0.686	0.471	35.59	***	−0.699	0.488	38.12	***
pH	−0.520	0.270	14.81	***	0.591	0.350	21.5	***
AP	−0.345	0.119	5.395	*	0.434	0.188	9.271	**
TN	−0.334	0.112	5.017	*	0.392	0.153	7.249	*
TP	0.370	0.137	6.342	*	−0.281	0.079	3.431	ns
SOC	−0.156	0.024	0.992	ns	0.220	0.048	2.03	ns
NO_3_^−^-N	0.066	0.004	0.175	ns	−0.015	0.000	0.009	ns
NH_4_^+^-N	−0.262	0.069	2.942	ns	0.281	0.079	3.437	ns
SWC	−0.181	0.033	1.357	ns	0.157	0.025	1.015	ns

ns represents not significant; *, **, and *** represents *p* < 0.05, *p* < 0.01, and *p* < 0.001, respectively.

## Data Availability

All the raw sequence data were deposited to the NCBI Sequence Read Archive (SRA) under project PRJNA876575.

## Supplementary Materials

The following supporting information can be downloaded at: https://www.mdpi.com/article/10.3390/microorganisms10112183/s1, Table S1. Location of sampling sites; Table S2. The statistics of metagenomic sequencing and assembly of microbial community in each soil sample; Table S3. Gene numbers assigned to organisms affiliated to different taxonomic levels; Table S4. The gene number and Shannon diversity index of microbial communities in each geographic site; Table S5. Relative abundances of organisms affiliated to different taxonomic levels; Table S6. The relative abundance of each COG category (level 1) in each sample; Table S7. Differential analysis of relative abundance of KEGG pathway (level 3) in microbial communities between hypersaline and saline soils; Table S8. Correlation of the KEGG pathways of the level 3 with EC; Table S9. The significantly differentiated genes involved in osmotic substance metabolism and ion transport between hypersaline and saline soil microbial communities; Figure S1. Relative abundance of COG level 1 categories genes in the saline and hypersaline communities; Figure S2. The genes annotated in each KEGG categories (A) and relative abundance of KEGG at level 1 in each sample (B); Figure S3. Relative abundance of KEGG level 1 (A) and level 2 categories genes (B) in the saline and hypersaline communities.
