# Supplementary material for: Metagenomic Insights into Microbial Community Structure, Function, and Salt Adaptation in Saline Soils of Arid Land, China
Source: Microorganisms. 2022 Nov 3;10(11):2183. doi: 10.3390/microorganisms10112183 (PMC9696928; doi:10.3390/microorganisms10112183)
Supplement: Supplementary file 1 [file microorganisms-10-02183-s001.zip › Table S1. Location of sampling sites.pdf]

Table S1. Location of sampling sites.

Geographic site index	Longitude and latitude		Altitude (m)	Location
ABH	83°14′E	44°37′N	172	Xinjiang, China
ALK	82°35′E	45°11′N	219	Xinjiang, China
BJT	85°6′E	445°40′N	208	Xinjiang, China
YSJ	86°14′E	44°59′N	290	Xinjiang, China
FKC	88°6′E	44°16′N	428	Xinjiang, China
CWP	87°52′E	43°31′N	1,039	Xinjiang, China
KMS	88°24′E	42°5′N	732	Xinjiang, China
HSB	86°42′E	42°16′N	997	Xinjiang, China
HJC	86°13′E	42°8′N	1,070	Xinjiang, China
ZNQ	86°19′E	41°50′N	984	Xinjiang, China
TMG	85°23′E	41°50′N	835	Xinjiang, China
KCB	83°9′E	41°28′N	916	Xinjiang, China
KPB	78°58′E	40°27′N	1,108	Xinjiang, China
BCB	78°30′E	39°53′N	1,069	Xinjiang, China
