# Supplementary material for: Metagenomic Insights into Microbial Community Structure, Function, and Salt Adaptation in Saline Soils of Arid Land, China
Source: Microorganisms. 2022 Nov 3;10(11):2183. doi: 10.3390/microorganisms10112183 (PMC9696928; doi:10.3390/microorganisms10112183)
Supplement: Supplementary file 1 [file microorganisms-10-02183-s001.zip › Table S4. The gene number and Shannon diversity index of microbial communities in each geo-graphic site.pdf]

Table S4. The gene number and Shannon diversity index of microbial communities in each geographic site.

**Samples**	**Gene numbers**	**Shannon index**	
		**genus**	**phylum**
HSB	3,138,591 ± 1,637,517	5.97 ± 0.28	1.75 ± 0.47
TMG	2,652,630 ± 296,530	5.83 ± 0.16	1.12 ± 0.20
HJC	4,242,615 ± 515,197	6.59 ± 0.28	2.05 ± 0.29
ZNQ	4,032,477 ± 338,532	5.51 ± 0.78	1.61 ± 0.29
KCB	2,044,519 ± 512,919	6.34 ± 0.23	1.69 ± 0.28
KMS	2,996,114 ± 106,623	5.04 ± 0.10	0.61 ± 0.18
BCB	2,981,616 ± 478,957	6.10 ± 0.23	1.62 ± 0.41
ABH	921,921 ± 31,897	7.26 ± 0.33	2.73 ± 0.18
ALK	2,300,766 ± 14,746	7.51 ± 0.26	3.07 ± 0.11
KPB	3,278,915 ± 470,829	6.72 ± 0.18	2.47 ± 0.11
BJT	3,533,709 ± 78,992	6.43 ± 0.10	2.18 ± 0.07
CWP	2,813,323 ± 143,735	7.03 ± 0.08	2.70 ± 0.06
FKC	2,697,890 ± 61,692	6.05 ± 0.04	2.13 ± 0.02
YSJ	3,009,692 ± 402,483	6.10 ± 0.09	2.19 ± 0.06
Total Mean	2,916,391 ± 920,460	6.32 ± 0.70	1.99 ± 0.67
Saline	2.544×10^{6} ± 850,819a	6.78 ± 0.593a	2.548 ± 0.081a
hypersline	3.07×10^{6} ± 1.08×10^{6}a	5.977 ± 0.115 b	1.577 ± 0.111b

Values in Mean ± Std. Deviation, n=3.

Different lowercase letters represents significant difference between saline and hypersaline soils ($p < 0.05$).
