# Supplementary figures and images for: Metagenomic Insights into Microbial Community Structure, Function, and Salt Adaptation in Saline Soils of Arid Land, China
Source: Microorganisms. 2022 Nov 3;10(11):2183. doi: 10.3390/microorganisms10112183 (PMC9696928; doi:10.3390/microorganisms10112183)

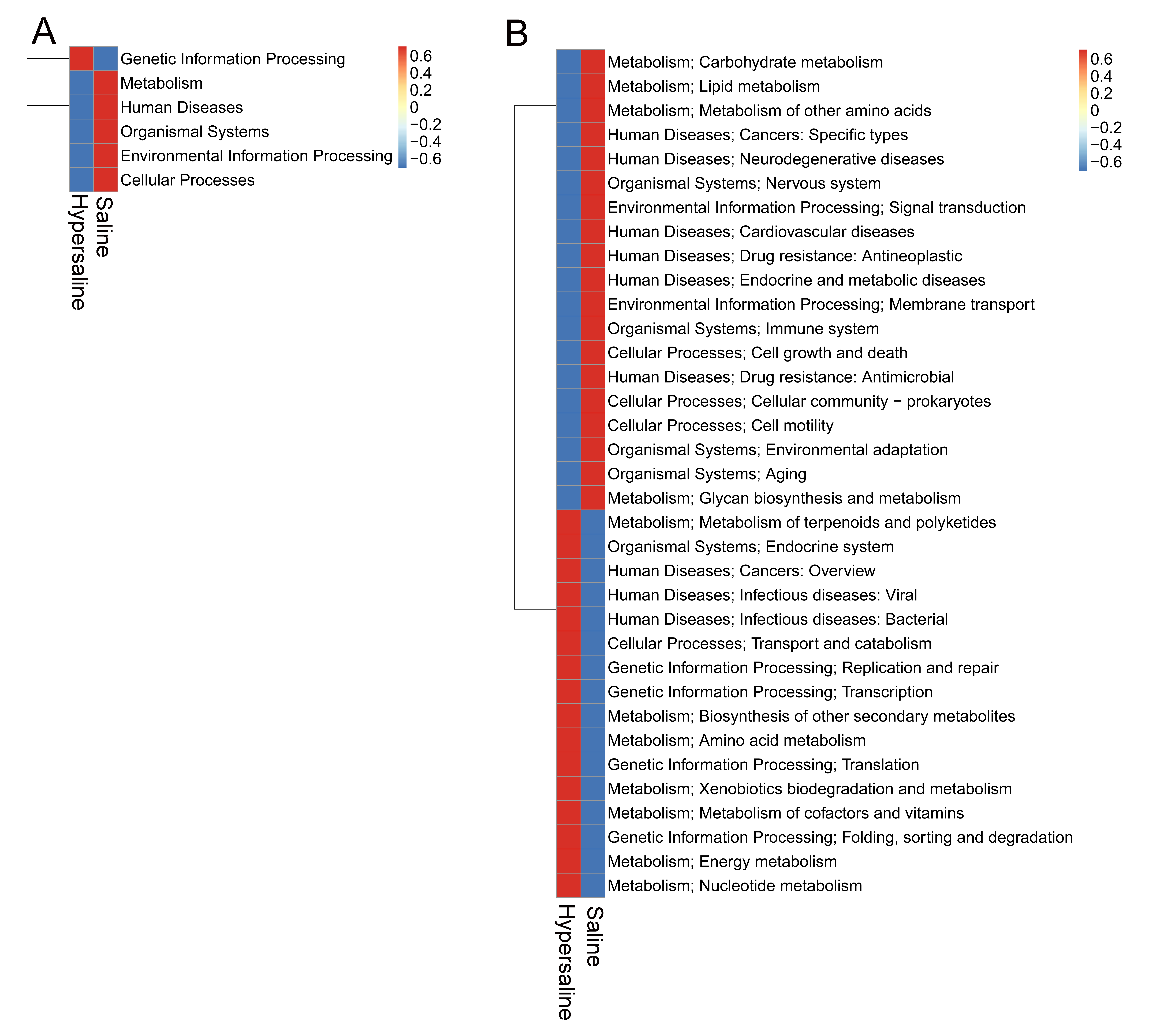

Supplement: Supplementary file 1 [file microorganisms-10-02183-s001.zip › Figure S3. Relative abundance of KEGG level 1 (A) and level 2 categories genes (B) in the saline and hypersaline communities.tif]
